# CD133^+^ cancer stem-like cells promote migration and invasion of salivary adenoid cystic carcinoma by inducing vasculogenic mimicry formation

**DOI:** 10.18632/oncotarget.8665

**Published:** 2016-04-09

**Authors:** Sha-sha Wang, Xiao-lei Gao, Xin Liu, Shi-yu Gao, Yun-long Fan, Ya-ping Jiang, Xiang-rui Ma, Jian Jiang, Hao Feng, Qian-ming Chen, Ya-jie Tang, Ya-ling Tang, Xin-hua Liang

**Affiliations:** ^1^ State Key Laboratory of Oral Diseases West China Hospital of Stomatology (Sichuan University), Chengdu Sichuan 610041, People's Republic of China; ^2^ Department of Oral Pathology, West China Hospital of Stomatology (Sichuan University), Chengdu Sichuan 610041, People's Republic of China; ^3^ Key Laboratory of Fermentation Engineering (Ministry of Education), Hubei University of Technology, Wuhan 430068, People's Republic of China; ^4^ Department of Oral and Maxillofacial Surgery, West China Hospital of Stomatology (Sichuan University), Chengdu Sichuan 610041, People's Republic of China

**Keywords:** adenoid cystic carcinoma (ACC), salivary gland, invasion, metastasis, vasculogenic mimicry (VM)

## Abstract

Cancer stem cells (CSCs) have gained much attention due to their roles in the invasion and metastasis of numerous kinds of human cancers. Here, we showed that the positive expression of CD133, the stemness marker, was positively associated with vasculogenic mimicry (VM) formation, local regional recurrence, distant metastasis and poorer prognosis in salivary adenoid cystic carcinoma (ACC) specimens. Compared with CD133^−^ ACC cells, CD133^+^ cancer stem-like cells had more migration and invasion capabilities, as well as more VM formation. The levels of endothelial cell marker VE-cadherin, MMP-2 and MMP-9 expression in CD133^+^ cancer stem-like cells and xenograft tumors of nude mice injected with CD133^+^ cells were significantly higher than those with CD133^−^ cells. The data indicated that CD133^+^ cancer stem-like cells might contribute to the migration and invasion of ACC through inducing VM formation.

## INTRODUCTION

Adenoid cystic carcinoma (ACC) is one of the most virulent human salivary gland cancers due to its tendency to hematogenous spread and invasion to distant tissues [1, 2]. Cancer stem cells (CSCs) has been shown to be the culprits for therapeutic resistance, recurrence and metastasis of tumors [3, 4]. CD133 protein was reported as both a molecular marker of poor prognosis in colorectal cancer, breast cancer and myeloid leukemia, and a marker of the CSCs population [5, 6]. Studies by Fujita group found overlapping populations of CD44 and CD133 markers in ACC [7]. However, whether CD133^+^ cells can be isolated and have more tumorigenic population in ACC or not has yet to be tested.

Vasculogenic mimicry (VM) was first discovered in 1999 in highly aggressive and metastatic melanoma cells, which could form patterned vascular channels lined externally by tumor cells without endothelial cells [8, 9]. Recently, accumulating evidences suggests that there are closely correlated between CSCs and VM formation in cancer [10–14]. Bussolati et al. [15] found that a number of the intra-tumor vessels were of human origin by injecting human breast CSCs into SCID mice in human breast cancer. Hendrix et al. [16] showed that vascular endothelial (VE)-cadherin was exclusively expressed in highly aggressive melanoma cells and was undetectable in the poorly aggressive tumor cells, suggesting their possible genetic reversion to an embryonic phenotype. However, the role and significance of CSCs in VM of ACC remains unclear.

In this study, we evaluated the correlations between CD133 and VM expression and the clinical-pathologic characteristics of 45 patients with ACC by immunohistochemical staining and histochemical double-staining methods. CD133+ cancer stem cells were isolated and identified, and we plated cells on Matrigel and observed whether CD133+ cells can give rise to VM. The migration and invasion of CD133+ and CD133– sorted cells were examined using wound-healing assay and Transwell invasion assays. The levels of endothelial cell marker VE-cadherin, matrix metalloproteinase (MMP)-2 and MMP-9 expression were measured in CD133+ cancer stem cells and xenograft tumors in nude mice injected with CD133+ and CD133– cells, respectively. Our data showed that CD133+ cancer stem-like cells might contribute to ACC progression and invasion via VM formation.

## RESULTS

### Correlation between expression of CD133 and clinic-pathological factors in ACC specimens

**Figure 1:**
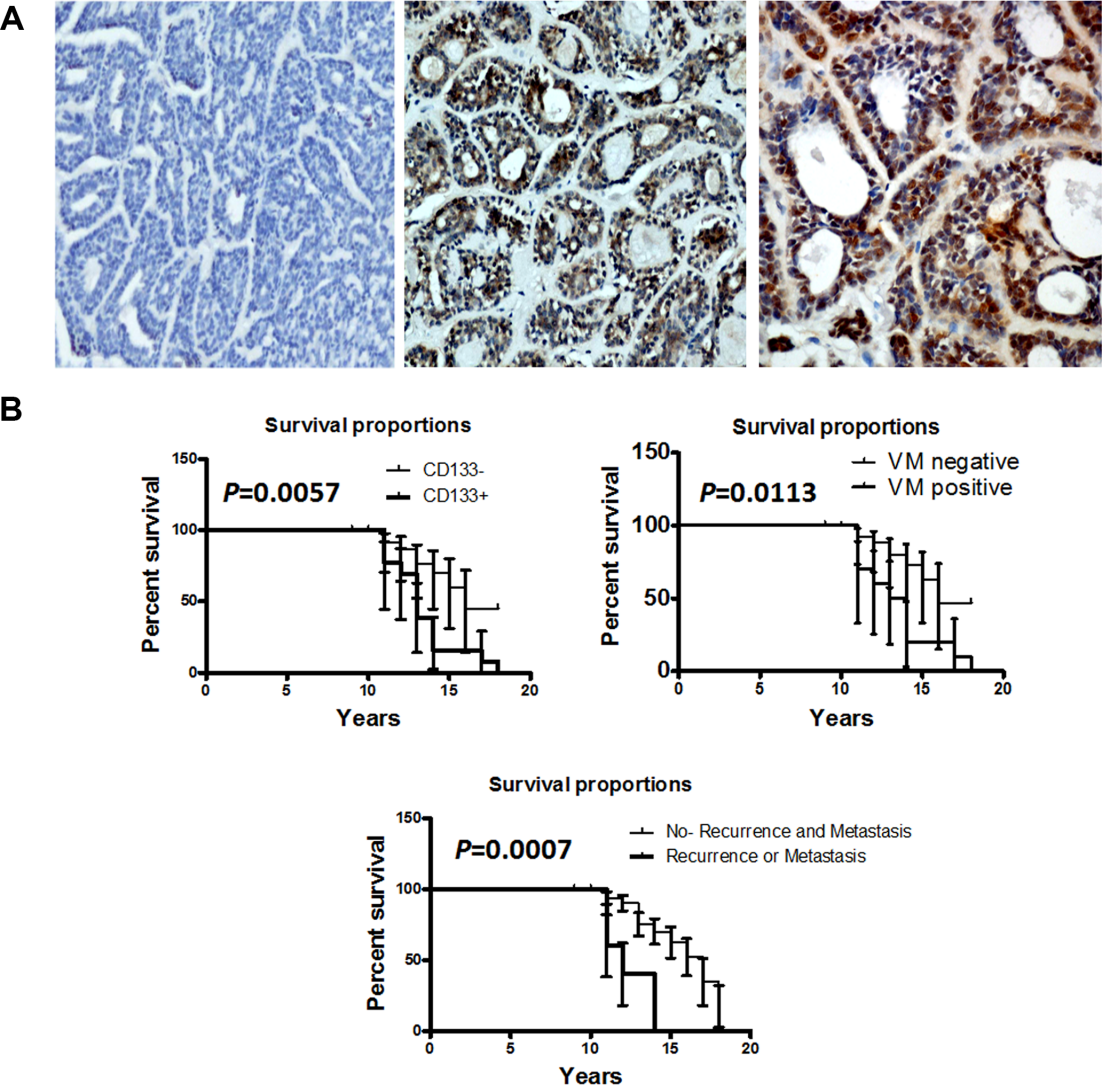
CD133 expression was associated with the prognosis of human ACC patients (**A**) Representative images of the immunohistochemical staining of CD133 in ACC samples and control group. A left, CD133 negative expression in human normal salivary tissue. C middle, CD133 in low positive tumor staining. C right, CD133 in strong positive tumor staining. Scale bar = 50 mm. (**B**) Kaplan-Meier survival analysis in patients with ACC. Overexpression of CD133 and VM in ACC was associated with a shorter overall survival of ACC patients (*P* = 0.0057 and *P* = 0.0113, respectively).

To evaluate the clinical relevance of CD133 expression in human ACC, we carried out immunohistochemical staining of CD133 in 45 human ACC samples. Representative immunohistochemical images were shown in Figure 1A. The staining of CD133 was confined to the membrane and cytoplasm of cancer cells. CD133 was expressed in 46.67% (21/45) specimens of salivary ACC, and not expressed in pleomorphic adenomas and normal salivary gland tissue. CD133 expression was significantly more common in salivary ACC than in pleomorphic adenoma and normal salivary gland tissue (*P* < 0.05). The level of CD133 expression therefore seems to correlate with malignant potential.

The relationship between the expression of CD133 and clinic-pathologic features of ACC was shown in Table 1. There was significant difference between minor salivary gland and major salivary gland (*P* = 0.0006). The positive expression of CD133 in 28.13% (9/32) of cases with tubular or cribriform pattern was much higher than in solid pattern (92.31%, 12/13) of ACC (*P* < 0.0001). The rate of CD133 positive expression in patients with local regional recurrence and distant metastasis was higher than without (*P* = 0.0018). However, there was no significant association of the CD133 positive expression status with age and sex of patients (*P* > 0.05). The patients with positive CD133 or metastasis had a poorer prognosis (a lower survival rate) than those with negative (*P* = 0.0057, *P* = 0.0007, respectively, Figure 1B). The univariate analysis showed that site, histological subtype, local regional recurrence and distant metastasis, and CD133 expression were significantly associated with patient survival (*P* < 0.05, Supplementary Table S1). Multivariate analysis using the Cox's proportional hazards model revealed that CD133 expression, local regional recurrence and distant metastasis were independent and significant prognostic factors in all patients (*P* < 0.05, Supplementary Table S2). These data confirmed CD133 as a novel prognostic molecular marker for ACC.

**Table 1:** Clinicopathological features of ACC patients and their association with CD133 and VM expression (n = 45)

Clinicopathological features	*n*	CD133 expression		*P* Value	VM		*P* Value
		Positive (*n* = 21)	Negative (*n* = 24)		Positive (*n* = 18)	Negative (*n* = 27)	
**Age**				0.8073			0.6190
< = 45	18	8	10		8	10	
> 45	27	13	14		10	17	
**Gender**				0.1606			0.5400
Female	25	14	11		11	14	
Male	20	7	13		7	13	
**Site**				0.0006			0.0004
Major salivary gland	29	8	21		6	23	
Minor salivary gland	16	13	3		12	4	
**T classification**				0.0015			0.0001
T1/T2	30	9	21		6	24	
T3/T4	15	12	3		12	3	
**Histological subtype**				< 0.0001			0.0107
Tubular/Cribiform	32	9	23		9	23	
Solid	13	12	1		9	4	
**Local regional recurrence and distant metastasis**				0.0018			0.0050
Yes	10	9	1		8	2	
No	35	12	23		10	25	

### CD133^+^ phenotype was positively associated with VM in adenoid cystic carcinoma specimens

**Figure 2:**
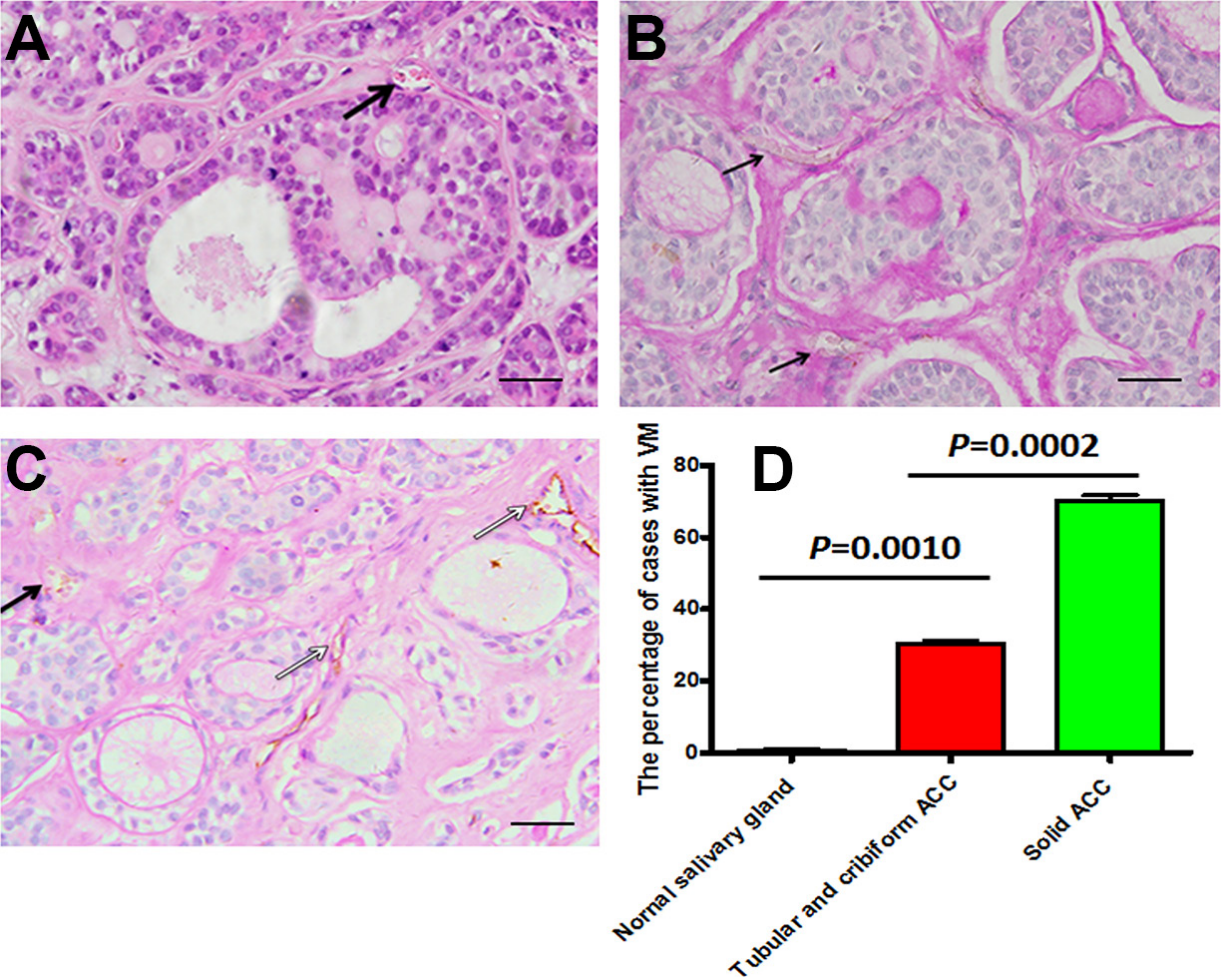
The presence of VM in salivary gland adenoid cystic carcinoma specimens (**A**) Typical blood vessels (black arrow) showed in ACC by HE. Scale bar = 50 mm. (**B**) VM (black arrow) in ACC tissue was identified by CD31 and PAS double staining. Arrow showed that adenoid cystic cancer cells formed extracellular matrix rich channels (PAS-positive), negative reaction for CD31 on their luminal surface and there was red cells in the channels. Scale bar = 50 mm. (**C**) VM (black arrow) in ACC and blood vessels (white arrow), which positive reaction for CD31 on their luminal surface and PAS-positive reaction in their wall. Scale bar = 50 mm. (**D**) The percentage of cases with VM in tubular or cribriform ACC, solid ACC and normal gland tissue.

We applied CD31 and PAS histochemical and immunohistochemical double staining to identify VM in human adenoid cystic carcinoma tissues. CD31-negative, PAS-positive vascular-like patterns containing red blood cells, which formed by cancer cells, were regarded as VM [17]. In this study, we found that the typical blood vessels showed positive reaction for CD31 on their luminal surface and PAS-positive reaction in their wall (Figure 2A, 2C). VM showed the PAS-positive tubular structures contained red blood cells but lined by CD31 negative cells on the luminal surface (Figure 2B, 2C). VM was detected in a total of 18 (40%) of 45 ACC patients, 9 (28.13%) of 32 tubular and cribriform pattern and 9 (69.23%) of 13 solid pattern (Figure 2D). The presence of VM was more in solid pattern of ACC specimens than in tubular or cribriform pattern (*P* = 0.0107). Reportedly, the solid subtype of ACC has the worst prognosis, with a survival of 34% at 10 years, in contrast to the 76% of the cribriform and the 100% of the tubular subtype [18]. The patients with VM expression had a poorer prognosis than those with negative (*P* = 0.0113, Figure 1B). The univariate analysis showed that VM expression were significantly associated with patient survival (Supplementary Table S1), however, multivariate analysis using the Cox's proportional hazards model revealed that VM expression was not an independent and significant prognostic factor in all patients (*P* > 0.05, Supplementary Table S2). This data indicated that adenoid cystic carcinoma cancer cells could mimic endothelial cells to form VM and VM has associated with the prognosis of ACC patients.

To investigate the relationship between CD133^+^ phenotype and VM in ACC specimens, we further used immunohistochemical staining to examine CD133^+^ expression in VM of ACC. Our results showed that adenoid cystic carcinoma cells with CD133^+^ staining can be found within VM structures, but also the cells with CD133^+^ staining can form VM channels containing red blood cells (Figure 2B, 2C, black arrow). CD133^+^/VM^+^ presented in 13 (28.89%) of 45 ACC, whereas CD133^−^/VM^−^ presented in 18 (40%) of 45 ACC. There was correlation between CD133 expression and VM in ACC cases by Chi-square (*P* < 0.05).

### CD133^+^ cancer stem-like cells promoted migration, invasion and VM of ACC

**Figure 3:**
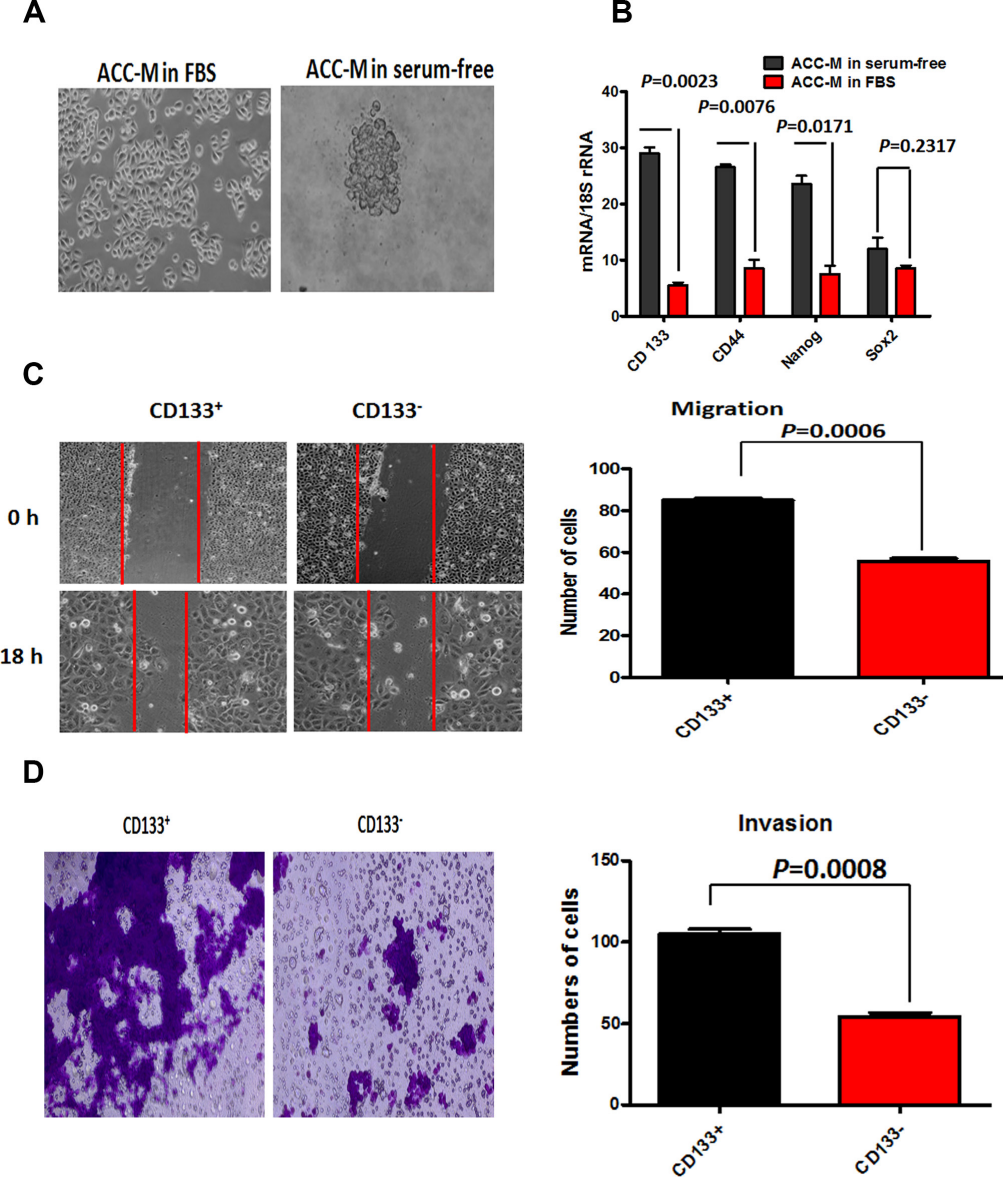
CD133+ cancer stem-like cells promoted ACC migration and invasion (**A**) ACC-M cells cultured in 10% FBS and serum-free conditions. Scale bar = 50 μm. (**B**) The relative mRNA expression of CD133, CD44, Sox2 and Nanog in ACC-M cells grown under 10% FBS conditions and serum-free. 18S was regarded as the housekeeping gene. The results showed that CD133, CD44 and Nanog of ACC-M cells in serum-free condition were quite higher than in FBS condition (*P* = 0.0023, 0.0076 and 0.0171, respectively), but the mRNA levels of Sox2 of ACC-M cells in serum-free condition was the same as in FBS condition (*P* = 0.2317). Each experiment was repeated 3 times. (**C** and **D**) The quantitative analysis of migration (C) and invasion (D) in CD133^+^ ACC-M and CD133^−^ ACC-M cells. Representative images of migrated and invaded cells were shown under inverted microscopy. The mean was derived from cell counts of 5 fields, and each experiment was repeated 3 times. Approximate 98% CD133^+^ ACC-M cells crossed the membrane and about 50% CD133^−^ ACC-M cells crossed the membrane in the transwell experiment. Scale bar = 50 μm.

To investigate the characteristic and function of CD133^+^ in ACC stem-like cell *in vitro*, we applied a basal condition (10% FBS) and a serum-free medium supplemented with EGF and bFGF to culture ACC-M cells. The results showed that ACC-M cell lines formed tumorspheres in serum-free condition, and only led to a layer of adherent confluent cells under FBS condition (Figure 3A). To exclude the possibility that cells may aggregate due to a high concentration of cells, one hundred ACC-M cells were used in culture medium. Tumorspheres could also be obtained in serum-free condition. These results showed that ACC-M cells could produce tumorsphere-like colonies in serum-free medium. Then, we carried out FCM to identify CD133^+^ ACC-M population with anti-human CD133 antibody. The results revealed that 10.1% ± 0.2% of cells in serum- free condition expressed the CD133 molecule, while 0.1% ± 0.4% of cells in FBS condition did. RT-PCR results showed that the mRNA levels of CD133, CD44 and Nanog of ACC-M cells in serum-free condition were quite higher than in FBS condition(*P* = 0.0023, 0.0076 and 0.0171, respectively), but the mRNA levels of Sox2 of ACC-M cells in serum-free condition was the same as in FBS condition (*P* = 0.2317, Figure 3B). Critically, we investigated the migration and invasion of CD133^+^ ACC-M and CD133^−^ ACC-M cells using wound-healing and transwell invasion assays. The results showed that the CD133^+^ ACC-M had more capability of migration and invasion than CD133^−^ ACC-M cells (Figure 3C, 3D), indicating that CD133^+^ ACC-M, cancer stem-like cells, promoted ACC migration and invasion. The similar results were shown in ACC-2, SACC-83, SACC-LM cell lines (Supplementary Figure S1). Herein, we purified and expanded the sorting CD133^+^ ACC-M cells by FCM as CD133^+^ cancer stem-like cells, and ACC-M cells in a basal condition as CD133^−^ cells for further study.

**Figure 4:**
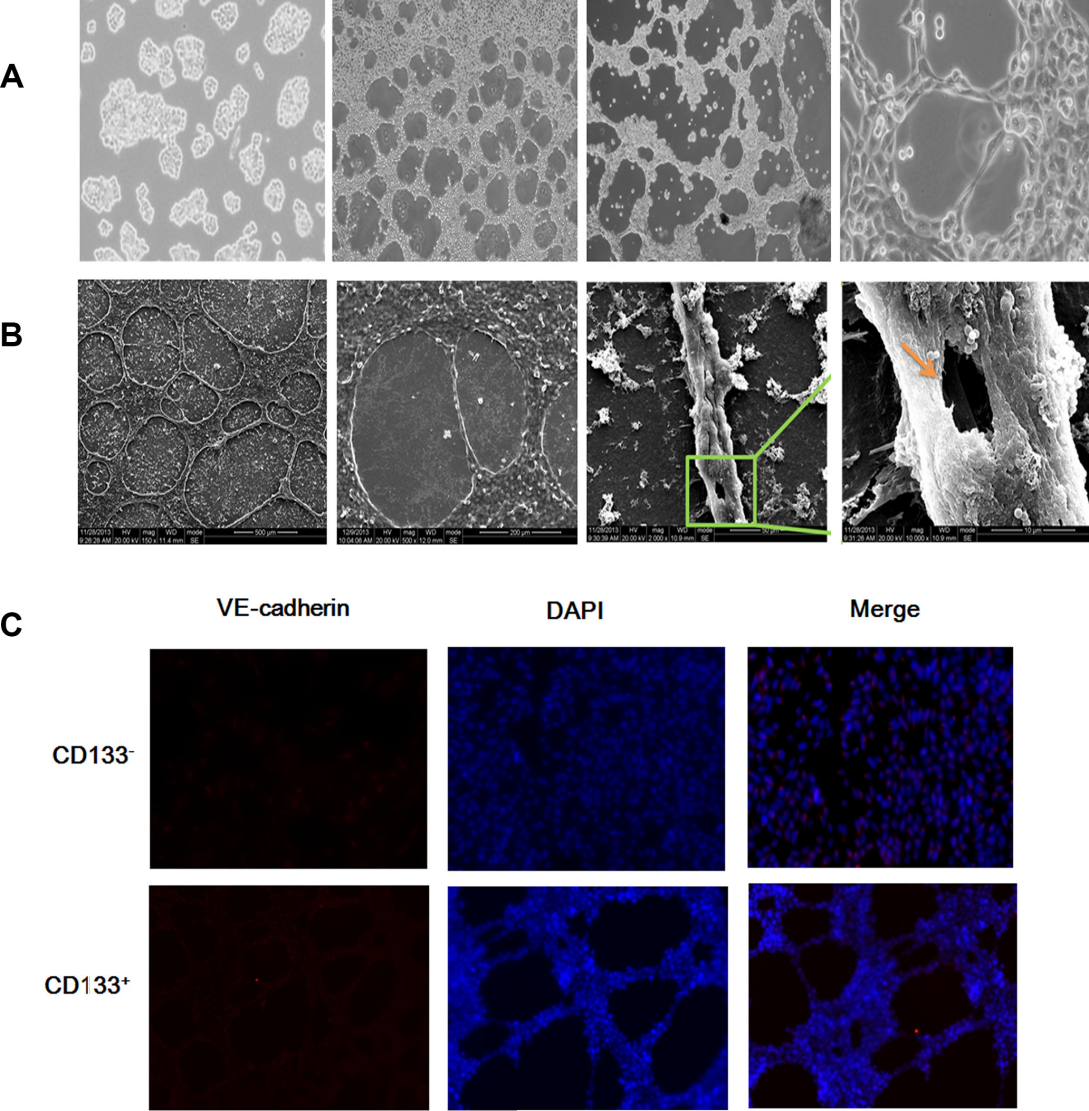
CD133^+^ cancer stem-like cells promoted VM formation in ACC (**A**) Representative images of the VM in ACC cells. From left to right, the figure showed was VM negative cells (control, 40×), VM in 24 h cells (40×), VM in 48 h cells (40×) and VM in the amplification of 48 h (200×). Scale bar = 50 μm. (**B**) Representative images of the VM in ACC cells under SEM. From left to right, the figures showed were amplified in turn and the values for the amplification were 150×, 500×, 2 000×, and 10 000×. (**C**) Representative images of the VM in ACC cells under fluorescence microscope. The picture showed that VM had been formed in CD133^+^ cells, not found in CD133^−^ cells. Scale bar = 50 μm.

To explore the association of CD133^+^ cancer stem-like cells with VM in ACC cells, we plated cells on Matrigel and observed that CD133^+^ cells began to give rise to tubular structures in < 24 h and produced very characterized microvascular channels by 48 h, whereas there was no tube formation in CD133^−^ cells (Figure 4A). Similarly, in Matrigel CD133^+^ cancer stem-like cells can generate patterns that consisted of a translucent tubular network under SEM and fluorescence microscope (Figure 4B, 4C), and CD133^−^ cells lacked this kind of tubular construct. These results provided support for the important role of CD133^+^ cancer stem-like cells in promoting VM formation of ACC cells.

### CD133^+^ cancer cells promoted expression of VE-cadherin, MMP-2 and MMP-9, and enhanced VM-like channel formation

Since CD133^+^ cells could form vessel-like structures within Matrigel, we hypothesize that they may have the potential to differentiate into endothelial cells and have the characteristic of endothelial cells. Then we examined the protein and mRNA levels of endothelial cell marker VE- cadherin, as well as MMP-2 and MMP-9, which are critical in cell plasticity and VM formation [19]. The result showed that CD133^+^ cancer stem-like cells significantly exhibited the increased protein and mRNA levels of VE- cadherin, MMP-2 and MMP-9 (*P* < 0.05), compared with CD133^−^ cells. The molecular change suggested that CD133^+^ cells in ACC might have endothelial cell phenotype.

**Figure 5:**
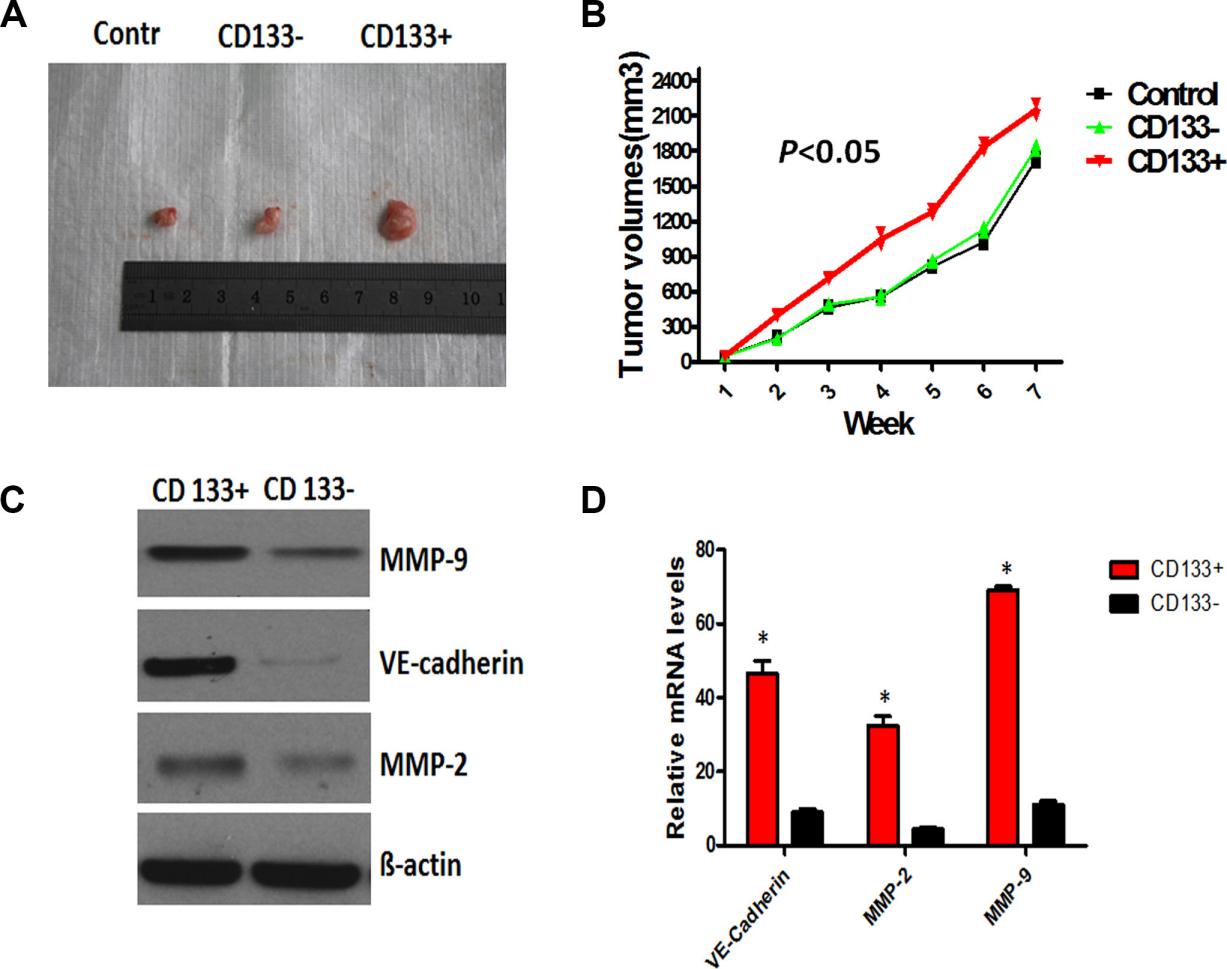
CD133^+^ cancer-like cells promoted the expression of VE-cadherin, MMP-2 and MMP-9, and enhanced VM- like channel formation (**A** and **B**) CD133^+^ and CD33^−^ ACC-M cells were subcutaneously injected into nude mice. Individual tumor volume was measured at the 7th week after injection and growth curve of xenograft tumors was shown. (**C** and **D**) Western blot and RT-PCR analysis of the protein and mRNA levels of VE-cadherin, MMP-2 and MMP-9 in CD133^+^, CD33^−^ and control group. ß-actin was used as the loading control in Western blot. GAPDH was regarded as the housekeeping gene for RT-PCR. Error bars represent the mean ± SD of triplicate experiments (*P < 0.05).

Then, we established xenograft in nude mice using CD133^+^ and CD133^−^ cells, and observed and calculated the tumor volume weekly. As shown in Figure 5A, 5B right, there was no difference of tumor volume observed between the CD133^+^ and CD133^−^ groups in the first week, however, the growth of tumor in the CD133^+^ group significantly increased since the 3th week, compared with the CD133^−^ group (*P* < 0.05). The relative quantification results showed that tumor cells transfected with CD133^+^ significantly up-regulated the protein and mRNA levels of VE-cadherin, MMP-2 and MMP-9, compared to those in CD133^−^ and control groups (Figure 5C, 5D), indicating that CD133^+^ cancer cells might facilitate the expression of VE-cadherin, MMP-2 and MMP-9, and enhanced VM-like channel formation.

## DISCUSSION

The growth of ACC is quite slow and the 5-year survival rates are very favorable at 70%–90%. However, the 5-year survival rates of patients with distant metastasis are as low as 20%, as well as how ACC gave rise to metastasis is still enigmatic to date [20]. With the development of cancer stem cells, recognized to play an important role in tumor relapse and resistance to chemotherapy, in this study, we hypothesized that CD133^+^ cancer stem-like cells involved in the migration and invasion of ACC.

There is some controversy on the association of the expression of CD133 with the prognosis of patients. In lung cancer and colorectal cancer, CD133 expression was shown to be an independent prognostic marker that correlated with low survival [21, 22]. However, Immervoll et al. [23] and Salnikov et al. [24] reported that there was no correlation between the expression of CD133 and patient survival of pancreatic ductal adenocarcinomas and non-small cell lung cancer (NSCLC), respectively. In this study, we showed that CD133 was identified in 46.67% of 45 ACC cases and the level of CD133 expression was associated with local regional recurrence, distant metastasis and poorer prognosis. We further showed that CD133^+^ cancer stem cells contribute to the migration and invasion of ACC, compared with the CD133^−^ cancer stem cells. These data indicated CD133 associated with the invasion and metastasis and functioned as a marker of prognosis in ACC.

Then, we try to address how CD133^+^ cancer stem-like cells contribute to the migration and invasion of ACC. VM, a new pattern of tumor microcirculation, is important for the growth and progression of tumors and CSCs play an important role in VM formation [37]. We first found VM formation in a total of 18 (40%) of 45 ACC patients and observed VM formation in ACC cells under invert microscope and SEM. This was consistent with the reports of Shirakawa group. In 2001, Shirakawa et al. [38] showed that the VM of inflammatory breast cancer (IBC) xenografts (WIBC-9) involved in hemodynamics that served to feed WIBC-9 cells, and this in turn suggests a connection between VM and angiogenesis. Then, this group exhibited VM without endothelial cells in regions of WIBC-9, revealing the expression of human-Flt-1 and human-Tie2 and the absence of human-CD31 [39]. Further, they confirmed that the existence of VM increased the likelihood of hematogenous metastases and was in inverse proportion to prognosis [40].

Importantly, the present study found that CD133^+^ phenotype was positively associated with VM in ACC specimens, and VM formation lacked in CD133^−^ cells. This was supported by many previous reports. Wu et al. [41] showed that the expression of CD133 was related to VM, lymph node metastasis, clinical stage, and prognosis of NSCLC patients. Lai et al. [42] reported that CD133^+^ cells drived tumor growth and the morphogenesis of a specialized perivascular niche by promoting VM in melanoma. These data indicated that CD133^+^ cancer stem-like cells might promote migration and invasion through inducing VM process.

VE-cadherin has always been regarded as an important role in the formation of VM. Hess et al. [43] showed that VE-cadherin and EphA2 acted in a coordinated manner as a key regulatory element in the process of VM formation in melanoma. Liu et al. [17] showed that CD133^+^ cancer stem cells up-regulated endothelial cell marker VE-cadherin in triple-negative breast cancer. This study demonstrated that the expression of VE-cadherin in CD133^+^ cancer stem-like cells was significantly higher than in CD133^−^ cells. Our data also showed that accompanied with the increasing of VE- cadherin, CD133^+^ cancer stem-like cells had the higher level of MMP-2 and MMP-9, [44, 45]. Therefore our results further demonstrated that CD133+ cancer stem cells might have the capacity of trans-differentiation and acquire endothelial cell phenotype and VE-cadherin expression, thus promoting VM.

Taken together, in this study we showed that CD133^+^ subpopulation in ACC was able to organize VM and VM might represent an important survival mechanism contributing to cancer migration and invasion. Further studies aimed at identifying the specific signaling pathways responsible for the ability of the CD133+ subset to generate VM.

## MATERIALS AND METHODS

### Ethics statement

The study of human specimens was approved by the Institutional Ethics Committee of the West China Medical Center, Sichuan University, China.

All animal studies were reviewed and approved by the Animal Care and Use Committee of the West China Medical Center, Sichuan University, China.

### Patients and specimens

The current study consisted of 45 ACC patients who had not undergone chemotherapy, hormone therapy or radiotherapy prior to surgery between 1996 and 2005. Formalin-fixed and paraffin-embedded samples from the patients were obtained from the Department of Oral Pathology, West China Hospital of Stomatology, Sichuan University. The sections from all cases were reviewed by two pathologists. Clinical and pathological variables were determined using well-established criteria (Table 1). The major gland included parotid gland, sublingual gland, submandibular gland, and the minor gland contained palatine gland, buccal, mouth floor, maxillary sinus, tongue, upper lip, mandibular ramus. In addition, 20 of pleomorphic adenoma, and 10 of normal salivary gland were included in this study. 20 of pleomorphic adenoma were from the paraffin-embedded samples of the patients at the Department of Oral Pathology, West China Hospital of Stomatology, Sichuan University between 1996 and 2005, and 10 of normal salivary gland were from human normal glands adjacent to salivary benign tumor. Every patient signed separate informed consent forms for sampling and molecular analysis. This study was approved by the Institutional Ethics Committee of the West China Medical Center, Sichuan University, China.

### Immunohistochemical (IHC) and histochemical double-staining methods

IHC was performed on 4-μm-cut representative sections by the streptavidin- peroxidase method followed as previously described [46]. The anti-CD133 antibody (Santa Cruz Biotechnology, 1:100) and anti-CD31 antibody (Beijing Zhongshan Biotechnology Limited Company, Peking, China, 1:40) primary antibody was used for the CD133 and CD 31 detection in cancer cells, respectively. PBS was used as the primary antibody for the negative controls. After IHC staining for the CD133 and CD 31, the sections were washed with running water for 5 min and incubated with periodic acid Schiff (PAS) for 15 min. Finally, all the sections were counterstained with hematoxylin, dehydrated and mounted.

Immunohistochemical results of CD133+ were analyzed by two independent pathologists (Wang SS, Liu X). The ratio of CD133+ cells was expressed as the percentage of 1000 tumor cells counted within 4–6 microscopic fields at ×400 magnification and semiquantitatively graded as follows: negative (0–9%), low positive (10–50%), high positive (> 50%).

CD31 staining was to identify endothelial cells, and any structure containing CD31-positive immunoreactivity was defined as a blood vessel. PAS staining was to determine the basement membranes of micro-vessels. VM structures were strictly defined as CD31-negative PAS-positive structures.

### Cell culture and 3D cultures

ACC cells lines (ACC-2, ACC-M, SACC-83, SACC-LM) were obtained from the State Key Laboratory of Oral Disease, Sichuan University. Cells were cultured in RPMI 1640 medium (Gibco) supplemented with 10% heat-inactivated FCS (Hyclone), 2 mmol/L L-glutamine, 25 mmol/L HEPES, and 100 units/mL penicillin and streptomycin in a humidified 5% CO_2_ atmosphere at 37°C.

Tumorsphere formation was performed in serum-free medium supplemented with 20 ng/mL human recombinant EGF and 10 ng/mL human recombinant bFGF (PeproTech). Single cell suspension was plated at 10,000 cells/mL, 2 ml per well on 6-well ultra-low attachment plates. Fresh medium was supplemented every 3 days. The mammospheres were counted at day 14.

Vasculogenic mimicry formation was tested using Matrigel *in vitro*. Twenty-four wells of culture plates were coated with Matrigel. The cells were trypsinized and suspended in the complete medium, plated onto the surface of Matrigel, and incubated at 37°C for 48 h. The numbers of tube-like structures were counted in a high-power magnification (×200). Cells were photographed using a phase contrast microscope.

### Flow cytometry (FCM)

The pretreated cells were harvested and washed twice with FCM buffer (PBS with 5% FBS and 0.1% NaN3). Cells were resuspended in PBS and incubated with PE-anti-human CD133 (BD Biosciences) or isotype control antibodies for 30 min at 4°C. CD133+ cells were sorted with a MoFlo XDP cell sorter (Beckman Coulter) and cultured in serum-free medium supplemented with 20 ng/mL human recombinant EGF and 10 ng/mL human recombinant bFGF.

### Western blot

Total proteins were isolated from the cultured monolayer cells with a total protein extraction kit (Keygen), and protein concentrations were detected by a bicinchoninic acid protein assay kit (Pierce). Membranes were blocked with 2% bovine serum albumin in TBS containing 0.1% Tween20 (TBST) at 37°C for 2 h and then incubated for 2 h respectively with primary antibody. ß-actin was used as the loading control. Bands were scanned using a densitometer (GS- 700, Bio- Rad Laboratories), and quantification was done using Quantity One 4.4.0 software. The antibodies of VE- cadherin (1:1000), MMP-2 (1:1000), MMP-9 (1:1000) (Abcam, UK) were used for Western blot.

### Quantitative real-time reverse transcriptase-PCR

Total RNA was isolated with TRIzol reagent (Invitrogen) and treated with RNase-free DNase I (Takara). PCR amplification of the cDNA template was done using Thunderbird SYBR qPCR mix (TOYOBO) on ABI PRISM 7300 sequence detection system (Applied Biosystems). GAPDH was regarded as the housekeeping gene. The relative expression level of the genes was calculated using the ΔΔCt method. The sequences of the primers were VE-cadherin Forward primer (5′–3′): CCCATCAGCTGCCCAGAA AATGAA, Reverse primer (5′–3′):CTGT CACCTTCAGCCATCCTGTTT; MMP-2 Forward primer (5′–3′): CTGACCCC CAGTCCTATCTG CC; Reverse primer (5′–3′):TGTTGGGAACGCCTGACT TCAG; MMP-9 Forward primer (5′–3′):CTTTGACA GCGACAAG AAGTGG; Reverse primer (5′–3′): GGCACTGAGGAATGATCTAAGC.

Real-time PCR for CD133, Sox2, Nanog and CD44 was performed and 18S was regarded as the housekeeping gene. The following primers were used: CD133 Forward primer (5′–3′): 5′-GAG TCG GAA ACT GGC AGA TAG CA-3′, Reverse primer (5′–3′): 5′-ACG CCT TGT CCT TGG TAG TGT TG-3; Sox2 Forward primer (5′–3′): 5′-GCC GAG TGGAAA CTT TTG TC-3′, Reverse primer (5′–3′): 5′-GTT CAT GTG CGC GTA ACT GT-3; Nanog Forward primer (5′–3′): 5′-CAG CTG TGT GTA CTC AAT GAT AGA TTT-3′, Reverse primer (5′–3′): 5′-CAA CTG GCC GAAGAA TAG CAA TGG TGT-3′; CD44 Forward primer (5′–3′): 5′-TGA ATA TAA CCT GCC GCT TTG-3′, Reverse primer (5′–3′): 5′-GCT TTC TCC ATC TGG GCC AT-3′. The gene expressions of CD133, Sox2, Nanog and CD44 were normalized to 18S assessed with standard primers and probes (Applied Biosystems, Warrington, UK). Each experiment was repeated at least 3 times.

### Wound-healing assay

CD133^+^ and CD133^−^ cells were plated in 6-well plates. When cells reached 80% confluence, the individual wells were wounded by scratching with a pipette tip and incubated with medium containing no FBS to 0, 18 h. Cells at the 2 time points were photographed under phase-contrast microscopy (×100) as previously described to compare the different numbers of the migration cells.

### Transwell invasion assays

*In vitro* cell invasion assays were performed with QCM^™^ 96-well cell invasion assay kit (Chemicon International, Temecula, CA, USA). 5 × 10^4^ cells were seeded into the top chamber coated with Matrigel (BD Biosciences). Complete medium was added to the bottom wells to stimulate invasion. After cells were incubated for 24–48 h, they were stained with 0.1% Crystal Violet. The cells that had invaded through Matrigel and reached to the reverse side were counted under a microscope in five pre-determined fields at a magnification of ×400. Each assay was performed in triplicate.

### Xenografts in nude mice

The fifteen nude mice (female, 6 weeks of age) were randomized and divided into three groups (CD133^+^ ACC-M, CD133^−^ ACC-M and Control). The control cells were SACC-LM. They fed in a room at a constant temperature (23 ± 2°C) and humidity (50–70%) with a 12-hr light-dark cycle, obtained from the Laboratory Animal Center of the West China Medical Center, Sichuan University (Chengdu, Sichuan, China). A total of 5 × 10^5^ cells (CD133^+^, CD133^−^ and the control) cells in 100 μL PBS were then injected subcutaneous on the back of nude mice. Tumor size was monitored by measuring diameters using vernier caliper weekly, and was calculated described previously [47]. Mice were euthanized and the tumors were removed at the 7th week. Harvested tumors were cut into 2 parts, one placed in liquid nitrogen and then frozen at −80°C for RT-PCR and Western blot, and the other fixed by 4% paraformaldehyde for H&E staining.

### Statistical analysis

The relationship between CD133 expression and clinicopathological factors was performed by the Chi-square test. Overall survival rate was examined using the Kaplan-Meier method and difference between groups was compared according to the log-rank test. A Cox proportional hazards model was used to identify prognostic variables. The data analysis was performed with the SPSS package (version 13.0). A value of *P* < 0.05 was considered significant.

## SUPPLEMENTARY MATERIALS FIGURES AND TABLES


