# Supplementary material for: CD133+ cancer stem-like cells promote migration and invasion of salivary adenoid cystic carcinoma by inducing vasculogenic mimicry formation
Source: Oncotarget. 2016 Apr 9;7(20):29051–62. doi: 10.18632/oncotarget.8665 (PMC5045377; doi:10.18632/oncotarget.8665)
Supplement: Supplementary file 1 [file oncotarget-07-29051-s001.pdf]

CD133+ cancer stem-like cells promote migration and invasion of salivary adenoid cystic carcinoma by inducing vasculogenic mimicry formation

Supplementary Materials

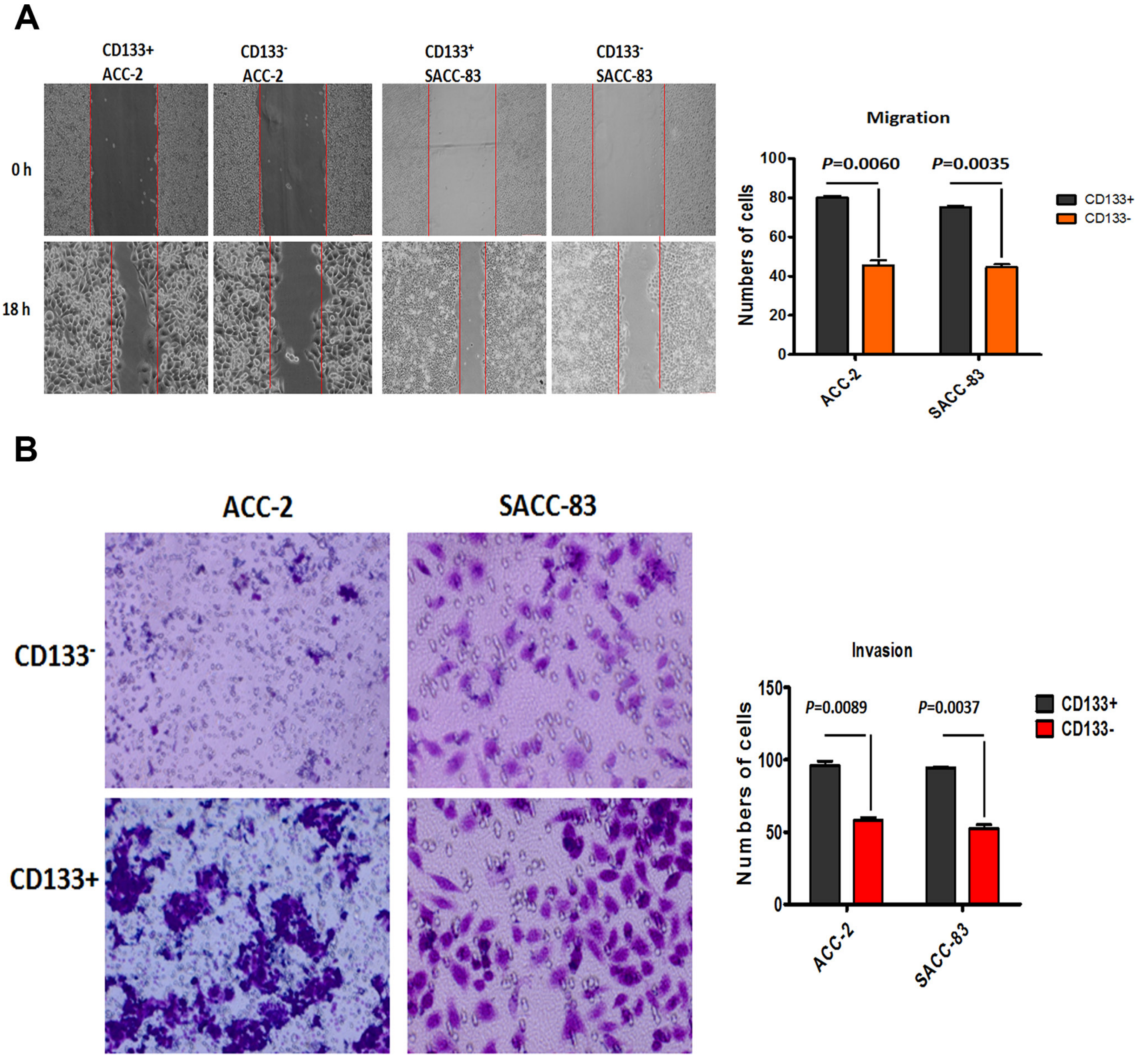

Supplementary Figure S1: CD133+ cancer stem-like cells promoted ACC migration and invasion. (**A** and **B**) The quantitative analysis of migration (A) and invasion (B) in CD133+ ACC-2 or SACC-83 and CD133- ACC-2 or SACC-83 cells. Representative images of migrated and invaded cells were shown under inverted microscopy. The mean was derived from cell counts of 5 fields, and each experiment was repeated 3 times. Approximate 95% CD133+ ACC-2 or SACC-83 cells crossed the membrane and about 50% CD133- ACC-2 or SACC-83 cells crossed the membrane in the Transwell experiment. Scale bar = 50 μm.

Supplementary Table S1: Univariable survival analysis of clinical and pathological data of 45 adenoid cystic carcinoma of salivary gland

Clinicopathological features	*n*	10 years Survival, %	*P* Value
**Age**			0.079
< = 45	18	35.2	
> 45	27	29.5	
**Gender**			0.094
Female	25	36.8	
Male	20	29.5	
**Site**			0.002
Major salivary gland	29	38.2	
Minor salivary gland	16	15.8	
**T classification**			0.061
T1/T2	30	31.4	
T3/T4	15	28.9	
**Histological subtype**			< 0.001
Tubular/Cribriform	32	42.9	
Solid	13	3.0	
**Local regional recurrence and distant metastasis**			< 0.001
Yes	10	17.2	
No	35	39.5	
**CD133 expression**			0.002
Positive	21	19.5	
Negative	24	40.2	
**VM expression**			0.006
Positive	18	23.3	
Negative	27	38.2	

Supplementary Table S2: Independent significant prognostic factors after cox multivariate survival analysis of adenoid cystic carcinomas of salivary gland

Variable	Categories	Relative risk (95% confidence interval)	*p*-Value
Regional recurrence and Distant metastasis	present	2.2 (1.4–4.4)	< 0.05
CD133 expression	positive	3.1 (2.0–5.1)	< 0.05
VM expression	positive		> 0.05
